# Prognostic alternative splicing signature reveals the landscape of immune infiltration in Pancreatic Cancer

**DOI:** 10.7150/jca.47877

**Published:** 2020-09-21

**Authors:** Lin Wang, Jia Bi, Xueping Li, Minjie Wei, Miao He, Lin Zhao

**Affiliations:** 1 Department of Pharmacology, School of Pharmacy, China Medical University, Shenyang, Liaoning Province, China.; 2 Liaoning Key Laboratory of Molecular Targeted Anti-tumor Drug Development and Evaluation; Liaoning Cancer immune peptide drug Engineering Technology Research Center; Key Laboratory of Precision Diagnosis and Treatment of Gastrointestinal Tumors, Ministry of Education; China Medical University, Shenyang, Liaoning Province, China.

**Keywords:** pancreatic cancer, alternative splicing, immune cells infiltration, prognosis, target therapy

## Abstract

**Background:** Pancreatic cancer (PC) is an aggressive cancer with worse survival in the world. Emerging evidence suggested that the imbalance of alternative splicing (AS) is a hallmark of cancer and indicated poor prognosis of patients. Genes-derived splicing events can produce neoepitopes for immunotherapy. However, the profound study of splicing profiling in PC is still elusive. We aimed to identification of novel prognostic signature across a comprehensive splicing landscape and reveal their relationship with tumor-infiltrating immune cells in pancreatic cancer microenvironment.

**Methods:** Based on integrated analysis of splicing profiling and clinical data, differentially splicing events were filtered out. Then, stepwise Cox regression analysis was applied to identify survival-related splicing events and construct prognostic signature. Functional enrichment analysis was performed to explore biology function. Kaplan-Meier curves and receiver operating characteristic (ROC) curves were performed to validate the predictive effect of predictive signature. We also verified the clinical value of prognostic signature under the influence of different clinical parameters. For deeper analysis, we evaluated the correlation between prognostic signature and infiltrating immune cells by CIBERSORT.

**Results:** According to systematic analyzing, a final six splicing events were identified and validated the good prognostic capability in entire TCGA dataset, validation set 1 and validation set 2 by Kaplan-Meier curves (*P* < 0.0001). The area under the curve (AUC) of ROC curves were also confirmed the high predictive efficiency of the prognostic signature in these three cohorts (AUC = 0.857, 0.895 and 0.788). In order to validate whether prognostic signature highlights a correlation between AS and immune contexture, CIBERSORT was performed to analyze the proportion of tumor-infiltrating immune cells in PC. Based on prognostic signature, we identified survival-related immune cells including CD8 T cells (*P* = 0.0111), activated CD4 memory T cells (*P* = 0.0329) and resting mast cells (*P* = 0.0352).

**Conclusion:** In conclusion, our study contribute to provide a promising prognostic signature based on six splicing events and revealed prognosis-related immune cells which indeed represented novel tumor drivers and provide potential targets for personalized therapeutic.

## Introduction

Pancreatic cancer is an aggressive disease with highly malignancy, poor prognosis and immune tolerance and remains one of the deadliest cancers in the world 1, 2. The 5-year survival rate for patients merely stands at 9% and the median survival time is 5-6 months 3. Tumor resection is the only possibility of cure, but the prognosis of patients is unfavorable in resected patients 4. In addition, clinical treatment results for patients are still undesirable because individual therapy strategies continue to have great challenges.

Gene expression profiling can provide evidence to identify survival-related prognostic biomarkers, or suitable therapeutic targets, in cancer 5. During the past few decades, lots of related studies have pointed out that the worst prognosis of PC is a difficult and strictly regulated process due to the increase of various gene alterations through the years. Recent whole-genome sequencing studies also pointed for genetic alterations in PC 6. It is understood that gene expression disorder serves an extremely important role in the course of cancer, which empowers PC to have the capacity of invasion, metastasize and reduced survival rate 7. However, many studies are mainly focusing gene variation in the process of cancer on transcript instead of pre-transcriptional level like splicing events. In particularly, systematic academic analysis of gene splicing alterations is limited in PC.

The wide studies of RNA-Seq revealed that alternative splicing (AS) is a highly pervasive mechanism which can produce different isoforms and affects more than 90% of human genes 8. The unbalanced expression of these isoforms between normal and tumor is changing in a wide range of extensive cancers contributing to tumorigenesis and the response to clinical therapy 9. Unbalanced expression of splicing variants is one of biological process of cancer cell changes 10. More and more reports suggested that differentially expression splicing variants is a hallmark of cancer 11. Splicing events of cancer have been involved in multiple oncological processes including angiogenesis, invasion and immune destruction 12. Similarly, splicing events are excessive in genes involved in immune cells of the tumor microenvironment. AS events are common in cancer-related immune cells, nearly 60 percent of genes previously unreported had frequent splicing isoforms in B cells or T lymphocytes 13. Thus, AS is important to achieve diversity and specialization of function in immune cells in order to improve the level of immune system to dynamically direct an effector response to pathogen invasion. Splicing events can provide some neoepitopes for the study of immune tolerance, proliferation and treatment of PC. Therefore, survival-related splicing events may be most significant in deciphering the underlying mechanisms of tumorigenesis, targeted immunotherapy and improving the prognosis of PC patients.

In this present study, based on integrated bioinformatics analysis, we build prognostic models which including six AS events. The AUC of the ROC curves and Kaplan-Meier (K-M) survival curves showed the valuable predictive efficacy of the model in PC. Aims to underlying the potential mechanisms between AS and 22 various immune cells, we performed CIBERSORT to analyze the proportion of tumor-infiltrating immune cells for this model in high-risk and low-risk patients. The results indicated that the AS model have strongly differential expression in many immune cells including memory B cells, naive B cells, CD8 T cells, macrophages M1, T cells regulatory (Tregs) and mast cells resting which are associated with survival in PC patients. Therefore, we discuss the relationship between AS and tumor-infiltrating immune cells for broaden the boundary of related cell-based immunotherapy.

In conclusion, we identified the prognosis of AS events by integrated bioinformatics analysis and understand in detail the potential relationship between AS and immune cells that regulate PC, which is a key step in developing immunotargeted therapy and improving the prognosis of PC patients.

## Materials and Methods

### Data curating process

Alternative splicing data of PC patients (n=176) were collected from SpliceSeq (http://bioinformatics.mdanderson.org/TCGASpliceSeq) which provide transcripts with variable splicing 14. RNA expression data (level 3, n=178) and clinical information of PC patients were downloaded from The Cancer Genome Atlas (TCGA) database (https://tcga-data.nci.nih.gov/) 15. After intersection of these patients, a total number of 175 patients with splicing data, RNA expression data and overall survival (OS) were included in the present study for further analysis. We also download an independent dataset with accession number of GSE28735 (n=90, 45 normal and 45 patients) from the Gene Expression Omnibus (GEO) database (https://www.ncbi.nlm.nih.gov/geo/) to serve as validation dataset. The platform of this dataset is GPL6244. These data were normalized by robust multi-array average (RMA) and log2-transformed which were used to validate the result.

### Identification of differentially AS events and construction AS related prognostic signature

In TCGA splice-seq, we analyzed the percent spliced in (*PSI*) value ranging from zero to one was calculated for each detected AS events in a gene to evaluate the mRNA splicing patterns in patients with PC 16. We performed a stringent filter of samples with *PSI* value ≥ 0.75. The overlapped AS events were visualized with Upset plot by using UpSetR package (R software version 3.5.2) which can depict the intersection of more than three sets and rank the intersections 17.

In order to identify differentially AS events between PC and normal tissues, the expression differences were characterized by absolute fold change (FC) over 2 and adjusted *P*-value < 0.05. Then, GO (Gene Ontology) and KEGG (Kyoto Encyclopedia of Genes and Genomes) pathway enrichment analysis of these AS events were performed by Bioconductor R package clusterProfler. In general, *P* ≤ 0.05 were represented significantly enriched pathways. Furthermore, univariate Cox regression analysis and multivariate Cox regression were performed to construct AS related prognostic model in PC patients by survival package in R (n=175). Finally, the prognostic signature, risk score, was calculated as followed:


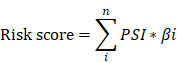


In the formula, *n, PSI* and *βi* represented the number, percent-spliced-in value and regression coefficient of splicing events, respectively.

### Validation of the prognostic signature

First, we performed differential expressed analysis of six genes in prognostic signature by GEO dataset (GSE28735) and The Human Protein Atlas database (HPA, https://www.proteinatlas.org/). Then, entire TCGA patients with PC (n=175) were randomly separated into two sets (TCGA validation set 1, n=88 and TCGA validation set 2, n=87). The prognostic signature was identified in entire TCGA data set and validated in both three sets. Based on risk score, patients were ranked into two (high/low) groups by the median cutoff value in three cohorts, respectively. K-M survival curves with Log-Rank test were applied to compare the OS effect of prognostic model in two risky sets. ROC curve was used to evaluate the predictive effect of prognostic marker by calculating 3-year survival with ROC package in R software. Likewise, we used stepwise Cox linear regression analysis to investigate the influence of clinical parameters in the prognostic signature by survival package in R and IBM SPSS 25.0 program.

### Validation the survival correlation between prognostic signature and tumor infiltrating immune cells in patients with PC

In order to explore the correlation between prognostic model and infiltrating immune cells, we need to perform CIBERSORT to identify the proportion of 22 different infiltrating immune cells in tumor immune microenvironment. CIBERSORT (http://cibersort.stanford.edu/), a computational framework which can provide detailed cell type abundance from tumor RNA profiles of intact tissues 18. In final, a total number of 113 patients with complete OS and risk score were included for further analysis. Based on risk score of prognostic signature, patients were separated into two (high/low) risk sets by the median value. Then, differently immune cell types between high versus low risky groups were tested by GraphPad Prism 8. Furthermore, univariate Cox linear regression or multivariate Cox linear regression was performed to evaluate the relationship between OS and infiltrating immune cells in cancer microenvironment. For survival-related immune cells, K-M survival curves and Log-Rank test were performed in PC patients.

### Statistical analysis

In this study, all data were used to determine independent prognostic factors which can predict patients' survival status by the R package (R software version 3.5.2). And the GraphPad Prism 8.0 software was performed to plot graphs containing K-M survival curve. All statistical analysis was used by IBM SPSS 25.0 program. Student *t* test (for equal variances) was performed and statistically significant *P*-value was set as ≤ 0.05 with the purpose of ensuring the reliability of the results.

## Results

### AS profiling and identification of differently survival-related AS events in PC

**Figure 1:**
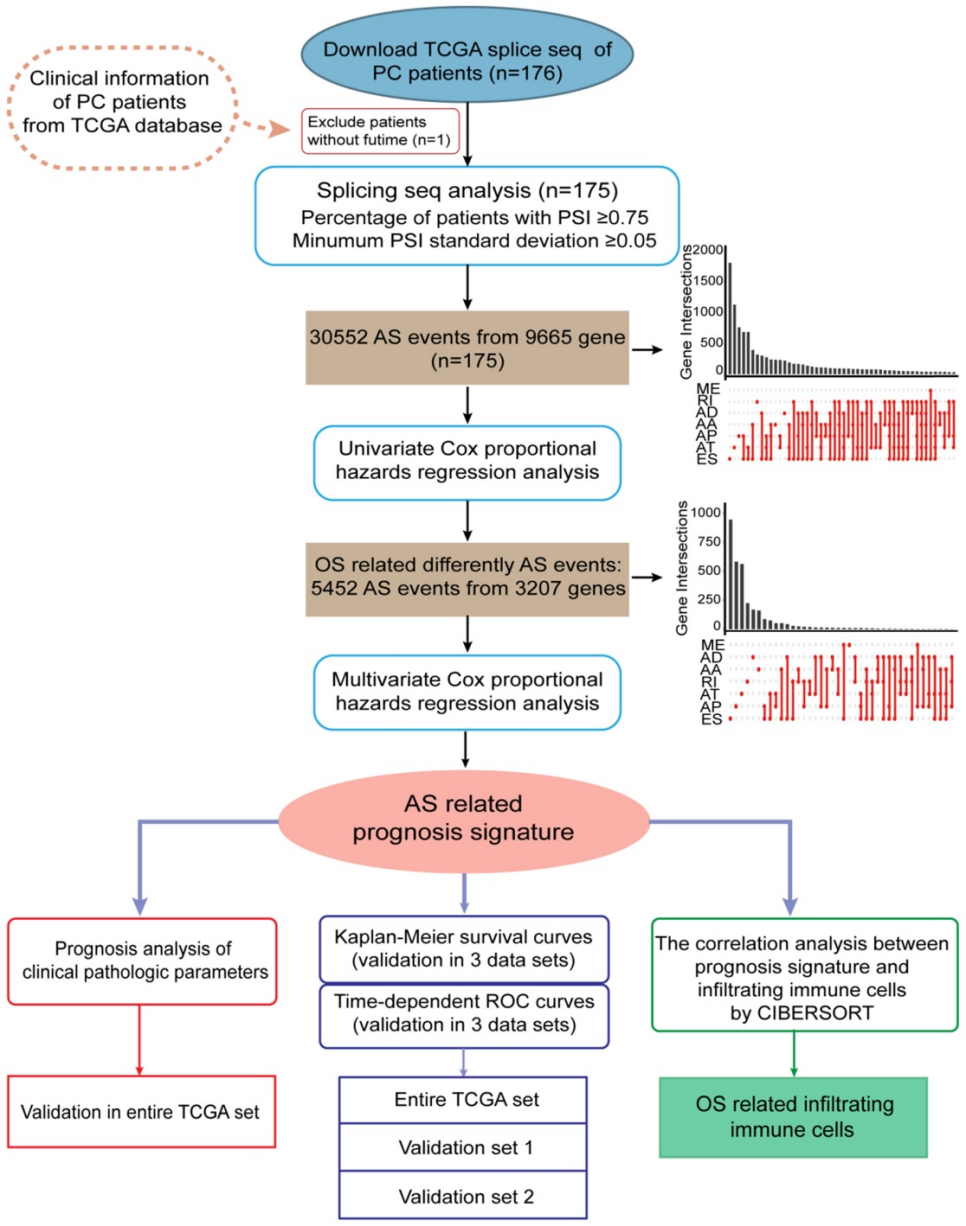
Flow diagram of data and analyses presented in this work.

The overall workflow in this study was summarized in Figure 1.

**Figure 2:**
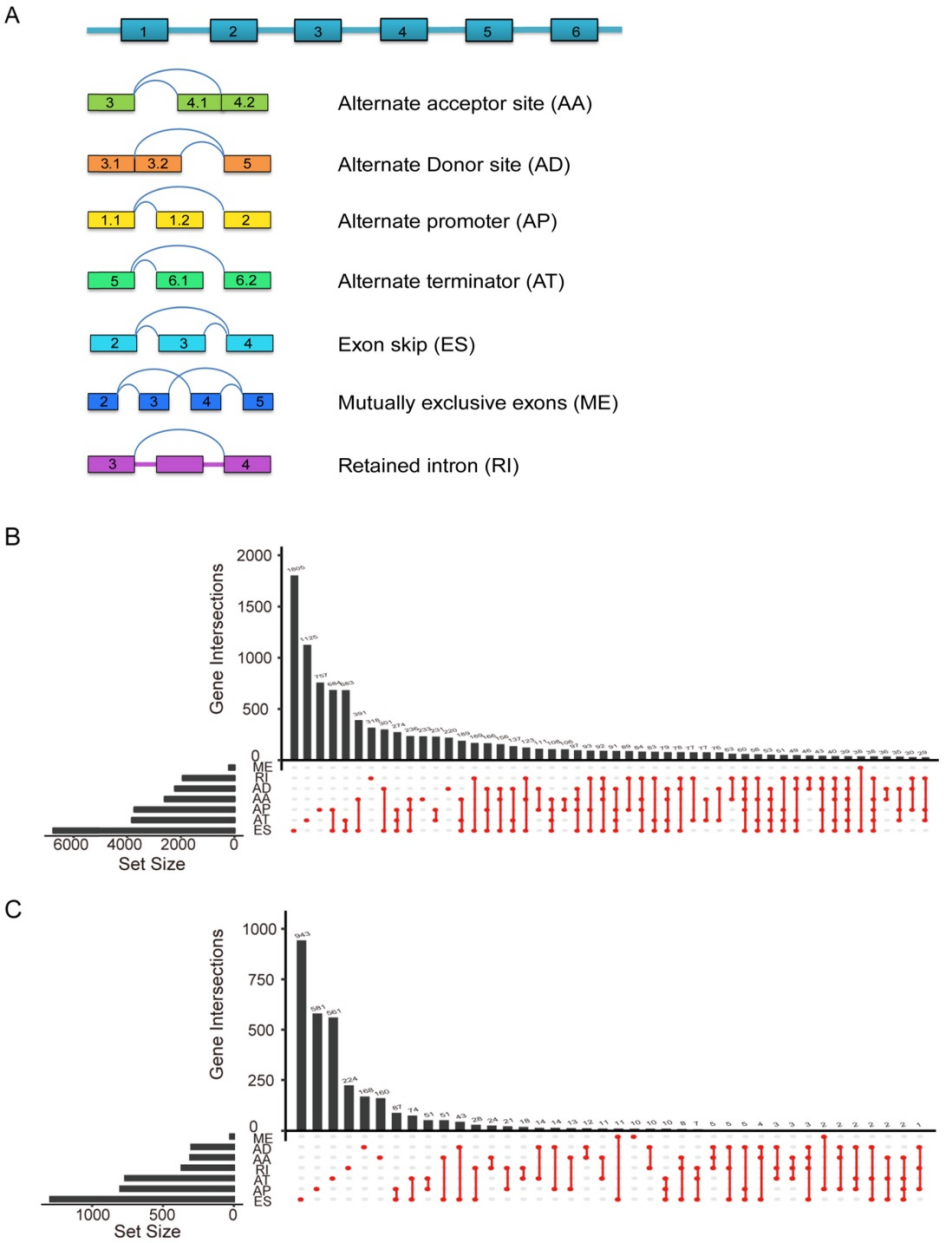
** Splicing events profiling in PC.** (**A**) Seven different AS types of genes including alternate acceptor site (AA), exon skip (ES), alternate donor site (AD), alternate terminator (AT), alternate promoter (AP), mutually exclusive exons (ME) and retained intron (RI). (**B**) Upset plot of interactions between different AS types in PC (n=175). One gene may have six AS types. (**C**) The Upset plot of different survival-associated AS types by performing univariate Cox regression in PC. One gene can produce more than four types survival-related AS events.

The integrated profiles of AS genes and events for 175 PC patients were analyzed using RNA-Seq data. The detailed clinical pathological data of patients from TCGA are summarized in Table 1. In total, we detected 30552 splicing events in 9665 genes by using SpliceSeq. All detected differently AS events can be classified into seven types: exon skip (ES), alternate acceptor site (AA), alternate promoter (AP), alternate donor site (AD), alternate terminator (AT), mutually exclusive exons (ME) and retained intron (RI), which were illustrated in Figure 2A. ES and AP events are the most frequent and the Upset plot shows the interaction numbers between genes and different AS class (Figure 2B). It is worthy that one gene may have up to several types of AS events and ES is the highest AS events in number instead of ME is the rarest.

Furthermore, in order to study the prognostic value of differently AS events, univariate Cox proportional hazards regression analysis were conducted in PC patients. As results, 5452 survival associated AS events were identified in 3207 differently expressed genes. UpSet plot of interactions between genes and OS associated AS events was shown in Figure 2C. One gene can produce more types survival-related AS events. AS shown in figure, seven AS events are associated with OS in PC patients and ES is the highest in number, but AA is the rarest. To demonstrate selected AS events have prognostic value, the top 20 most significant prognosis-associated AS genes of each types are illustrated in Supplementary Figure S1A-G. As shown in results, we identified the prognostic signature including AP in UBA1, S100A13, SH3KBP1 and COPS7A, ES in GSE1 and AT in NISCH are included.

In addition, it was evident that AS is a RNA processing pathway, which can change the function of protein. For a deeper understanding of the potential biological significant of the survival-related AS events, GO categories (Supplementary Figure S2A) and KEGG pathway (Supplementary Figure S2B) were performed. In GO analysis, there are we detected that AS events are essential in mRNA splicing which mainly enriched in regulation of mRNA processing, metabolic process, cytoplasmic region, cell-substrate adhesion junction and actin binding. The significant KEGG pathways of the AS events are including regulation of actin cytoskeleton and ErbB signaling pathway. Taken together, above results represented that genes with AS events play a valuable role in the biological process of PC.

### Construction of survival-associated AS prognostic model

**Figure 3:**
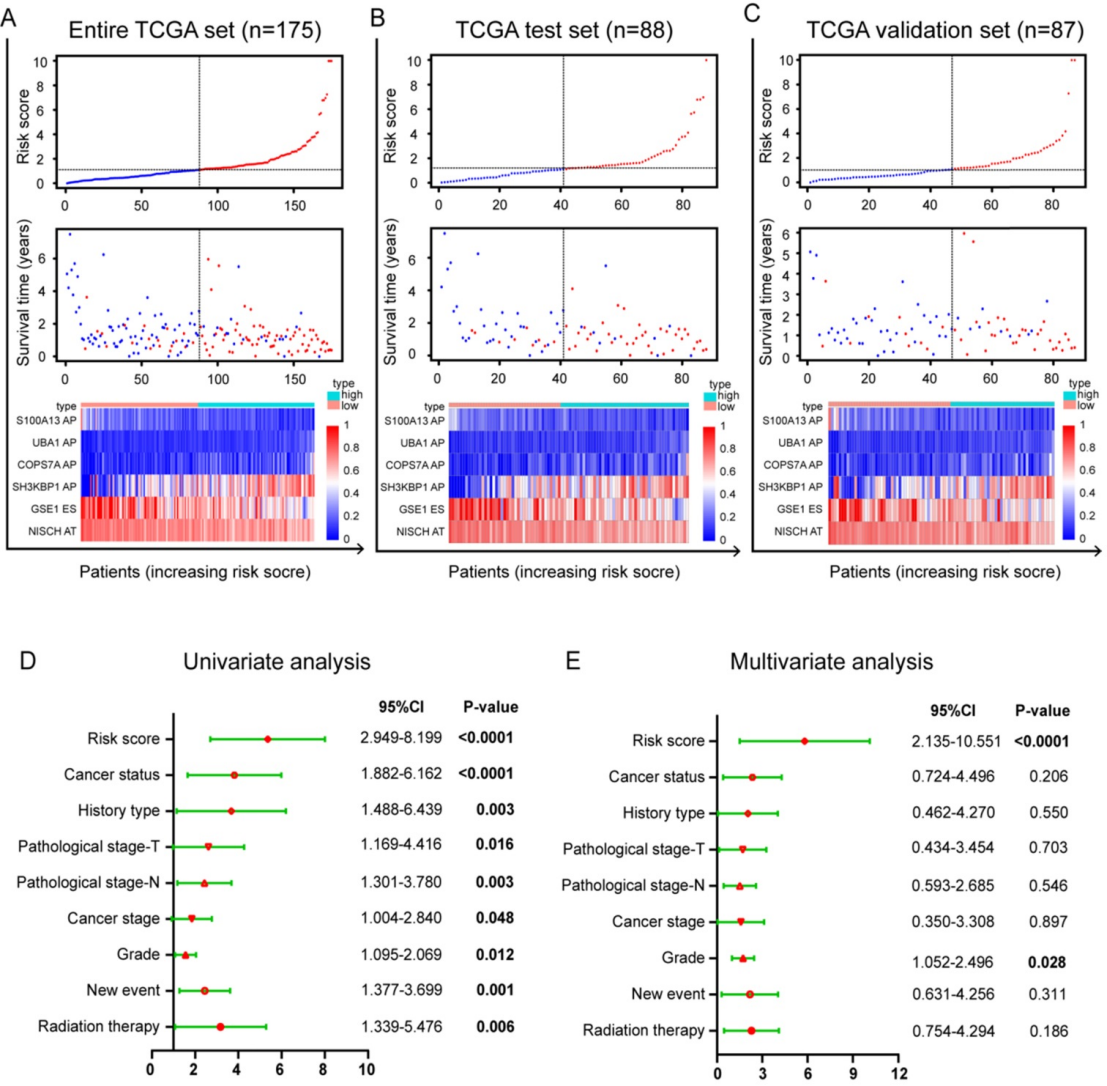
** Identification of prognostic AS model and construction a prognostic risk score system in PC.** Distribution of risk stratification based on prognostic signature was validated in three cohorts including (**A**) entire TCGA set (n=175), (**B**) TCGA test set (n=88) and (**C**) TCGA validation set (n=87). The top part displays and sorts the patients' survival data based on risk score, the middle part shows the risk score's distribution curve and the bottom part (heat map) presents of the *PSI*s value of each prognostic signature. Forest plot visualizing hazard ratios of significantly survival-related clinical pathological parameters including cancer status, history type, pathological stage T, pathological stage N, cancer stage, grade, new event, radiation therapy by performing univariate (**D**) and multivariate (**E**) Cox regression analysis. A two‐sided Log‐Rank and Wilcoxon *P*<0.05 were considered significant.

Lasso regression (removing genes with high correlation) was applied on differently OS-related AS events after univariate Cox survival analyses and then further multivariate Cox hazards regression analyses was carried out to determine independent prognostic indicators in PC. Finally, we obtained six genes with AS events (AP in UBA1, S100A13, SH3KBP1 and COPS7A, ES in GSE1 and AT in NISCH) could be recognized as an independent prognostic risk scoring system in PC patients (n=175, Supplementary Table S1). Based on the formula of risk score, patients were divided into two (low and high) risk groups using the median risk value in entire TCGA data set (n=175, Figure 3A), TCGA validation set 1 (n=88, Figure 3B) and TCGA validation set 2 (n=87, Figure 3C), respectively. These results showed that risk score may exhibit much more prognostic efficiency.

Furthermore, we performed univariate and multivariate survival tests in order to prove the predictive ability of the prognostic signature and different clinical pathological parameters including cancer status, history type, pathological stage-T, pathological stage-N, cancer stage, grade, new event and radiation therapy (Table 2). Univariate survival analyses showed that risk score and the above mentioned clinical factors can be prognostic biomarker in PC patients (n=175, Figure 3D). As showed in Figure 3E, risk score (*P*<0.0001) and grade (*P*=0.028) were independent prognostic indicators with significant differences for PC patients in multivariate survival analysis. Although other clinical indicators in multivariate survival analysis were less powerful, they still have potential value in clinical application. It is notable that risk score (*P*<0.0001) had the strongest predictive ability among these indicators.

### Validation of the prognostic signature in PC patients

**Figure 4:**
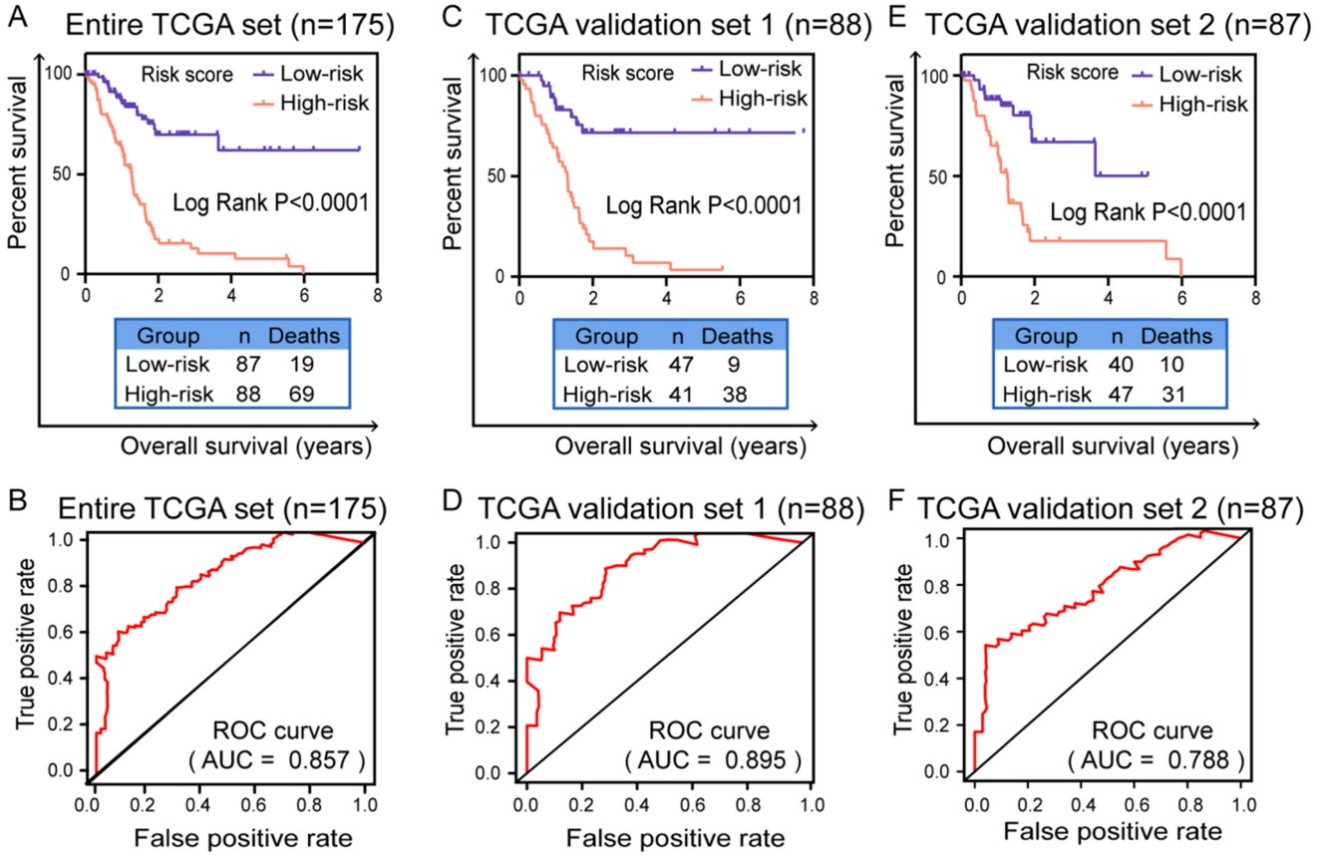
** Validation of prognostic model for PC patients with Kaplan-Meier survival curves and ROC curves.** Kaplan-Meier survival curves of prognostic signature for PC patients in (**A**) entire TCGA set (n=175), (**C**) TCGA test set (n=88) and (**E**) TCGA validation set (n=87). A two‐sided Log‐Rank *P*<0.05 were considered significant. ROC curves were applied to validate prognostic efficiency of prognostic biomarker for PC patients at 3 years in (**B**) entire TCGA set (n=175), (**D**) TCGA test set (n=88) and (**F**) TCGA validation set (n=87). Abbreviation: ROC: receiver operating characteristic.

In order to verify the expression of six genes in prognostic signature is significantly different between normal and patients, we conducted the validation in GSE28735 and HPA database, respectively. As shown in Supplementary Figure S3A, all six genes were differentially expressed in GSE28735 and the difference was statistically significant (*P*<0.05). Immunohistochemical results (Supplementary Figure S3B) in HPA database also showed that the moderate or high staining intensity and cell quantity (>75%) of these four genes (UBA1, S100A13, SH3KBP1 and NISCH) in PC tissues contrasted sharply with the lack or low staining intensity and small quantity (<75%) in normal tissues, while COPS7A presented high expression level in both normal and tumor tissue. For GSE1, the staining intensity and quantity in normal tissue is higher than which in tumor tissue. These results indicated that the expression of most of the six mRNAs is significantly different between normal and PC tissues. Furthermore, in order to validate the predictive capability of prognostic signature, survival curves were generated by K-M survival analysis in three cohorts. The time-dependent ROC curves were applied to assess the prognostic efficiency and accuracy of patients with this prognostic model. In entire TCGA data set (n=175), the K-M survival curve showed that the prognostic signature indeed can well distinguish patients into high or low survival rate (*P*<0.0001, Figure 4A). ROC curve (AUC) for predicting patients survival confirmed that the identified prognostic signature has the robust efficiency to predict the OS for PC patients (AUC=0.857, Figure 4B). In TCGA validation set 1 (n=88), the K-M curve (*P*<0.0001, Figure 4C) and ROC curve (AUC=0.895, Figure 4D) also showed that the prognostic signature indeed have robust predictive ability in PC patients. In TCGA validation set 2 (n=87), the K-M survival curve also showed evident gaps between low-risk and high-risk patients (*P*<0.0001, Figure 4E) and the ROC curve (AUC=0.788, Figure 4F) again validated that the prediction model can be good prognostic indicator in patients with PC.

### Validation independent prognostic indicator of the six-AS events from clinical pathological factors in entire TCGA set

**Figure 5:**
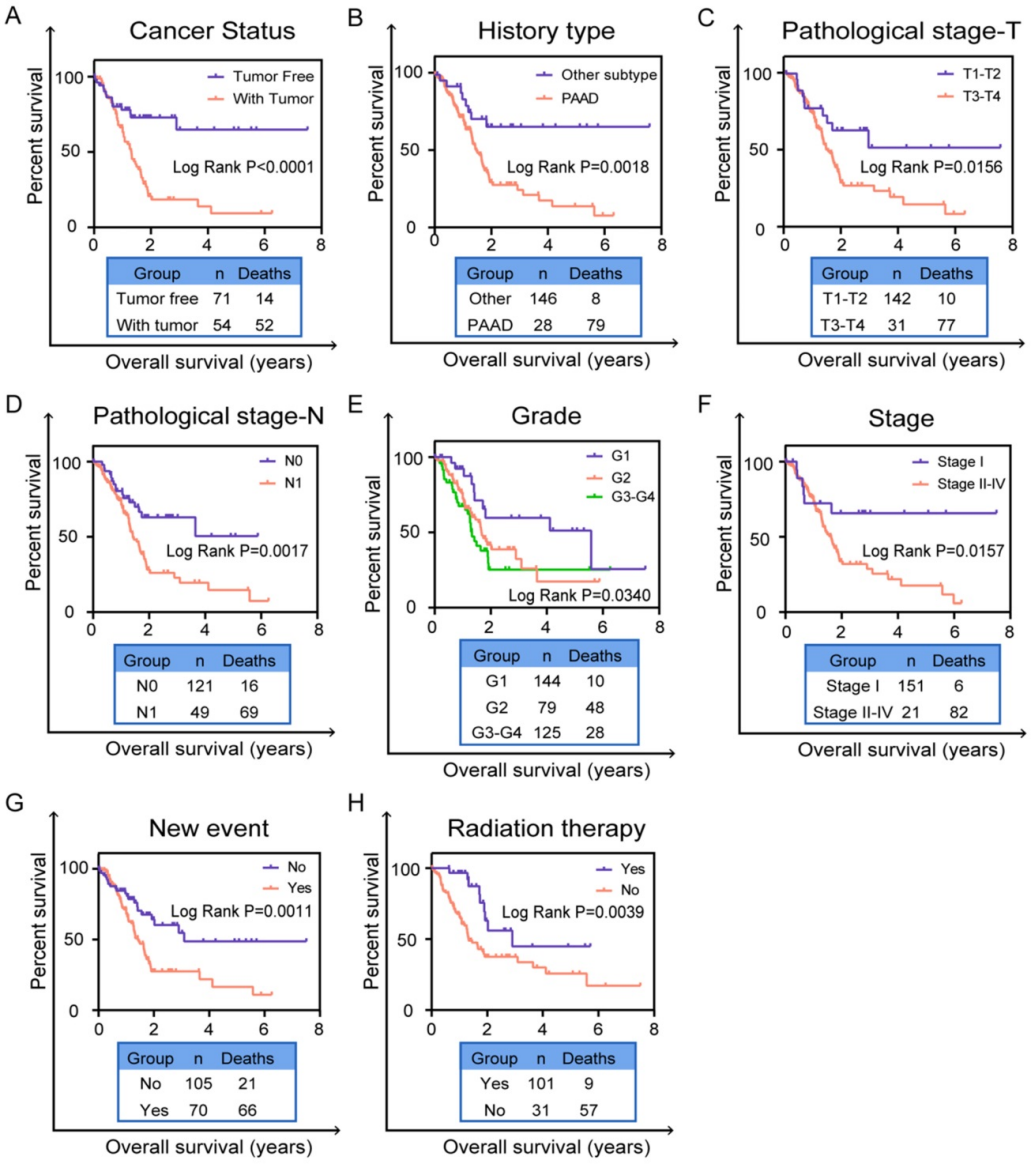
** Validation prognostic indicators in clinical pathological parameters in patients.** The Kaplan-Meier survival curves of prognostic biomarker in clinical pathological parameters including (**A**) cancer status, (**B**) history type, (**C**) pathological stage T, (**D**) pathological stage N, (**E**) grade, (**F**) cancer stage, (**G**) new event, (**H**) radiation therapy. A two‐sided Log‐Rank and Wilcoxon *P*<0.05 were considered significant.

Based on the previous univariate survival analysis, we know that clinical parameters were effective prognostic predictors in patients with PC. Thus, we performed the K-M survival curves to validate the predictive value of clinical parameters including cancer status (*P*<0.0001, Figure 5A), history type (*P*=0.0018, Figure 5B), pathological stage-T (*P*=0.0156, Figure 5C), pathological stage-N (*P*=0.0017, Figure 5D), grade (*P*=0.0340, Figure 5E), cancer stage (*P*=0.0157, Figure 5F), new event (*P*=0.0011, Figure 5G) and radiation therapy (*P*=0.0039, Figure 5H). Obviously, patients who were with tumor, pancreatic adenocarcinoma (PAAD), in pathological stage-(T3-T4), in pathological stage-N1, in grade-(G2-G4), in stage II-IV, with new event and without radiation therapy had worse prognosis in entire TCGA set. These results further validated that these clinical indicators indeed have good prognostic value in patients of PC.

**Figure 6:**
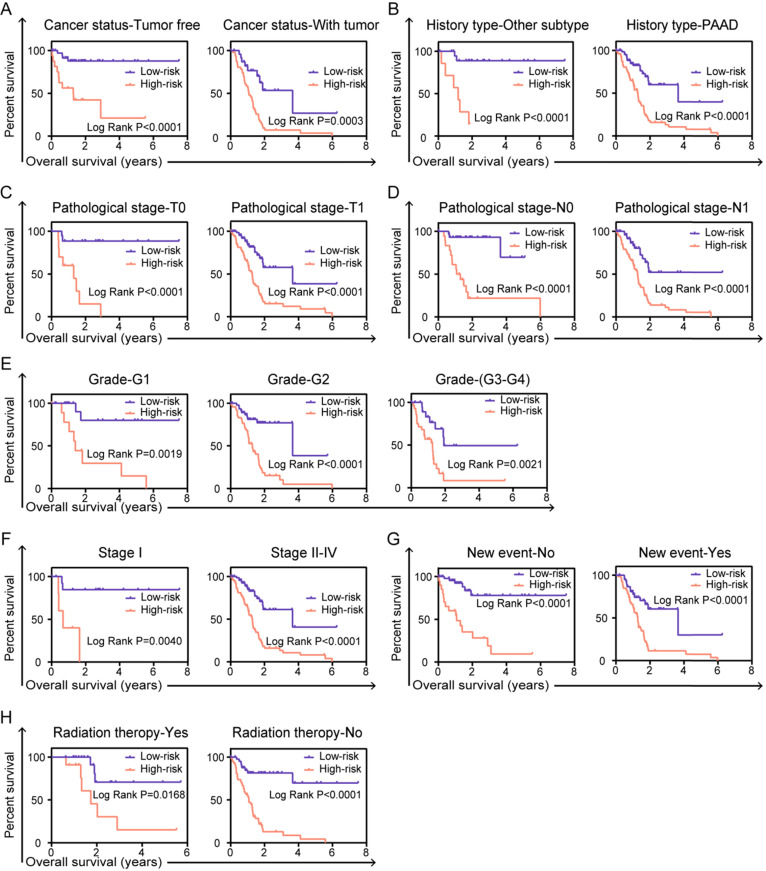
** Stratified analyses and validate prognostic signature in clinical pathological parameters for PC patients.** The Kaplan-Meier survival curves of prognostic biomarker in the subtype of (**A**) cancer status, (**B**) history type, (**C**) pathological stage T, (**D**) pathological stage N, (**E**) grade, (**F**) cancer stage, (**G**) new event, (**H**) radiation therapy. A two‐sided Log‐Rank and Wilcoxon *P*<0.05 were considered significant.

In order to fully understand the clinical value of prognostic signature, stratified survival analysis was performed to validate whether the prognostic model is indeed significant in clinical application. For clinical parameters of cancer status (tumor free or with tumor, Figure 6A), history type (other types or PAAD, Figure 6B), pathological stage-T (T0 or T1, Figure 6C), pathological stage-N (N0 or N1, Figure 6D), grade (G1 or G2-G4, Figure 6E), cancer stage (stage I or stage II-IV, Figure 6F), new event (no or yes, Figure 6G) and radiation therapy (yes or no, Figure 6H), risk score based on the integrated six-AS events signature can be confirmed as independent prognostic indicator and have great value in clinical application of patients in PC.

### Revealing the relationship between prognostic signature and tumor-infiltrating immune cells in tumor microenvironment

**Figure 7:**
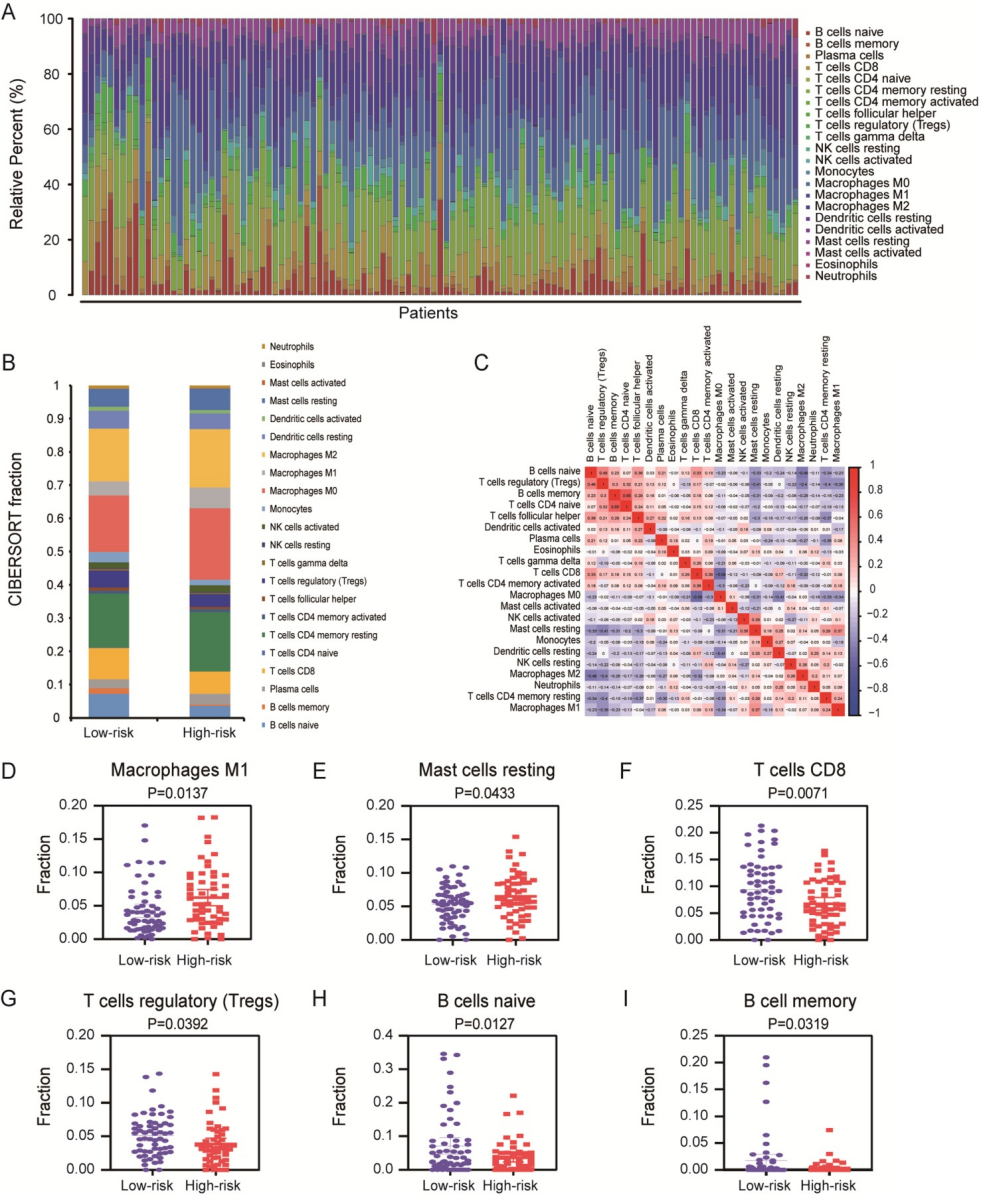
** Assessment of prognostic signature from 22 immune infiltration cells in PC.** (**A**) The summary of 22 immune cells' subpopulations in 113 samples. (**B**) Composition of infiltrating immune cells in different risk (high/low) groups. (**C**) Correlation analysis of all 22 immune infiltrating cells. Differently fractions of (**D**) macrophages M1, (**E**) resting mast cells, (**F**) CD8 T cells, (**G**) T cells regulatory, (**H**) naive B cells and (**I**) memory B cells in low-risk and high-risk groups, respectively. A two‐sided Log‐Rank and Wilcoxon *P*<0.05 were considered significant.

**Figure 8:**
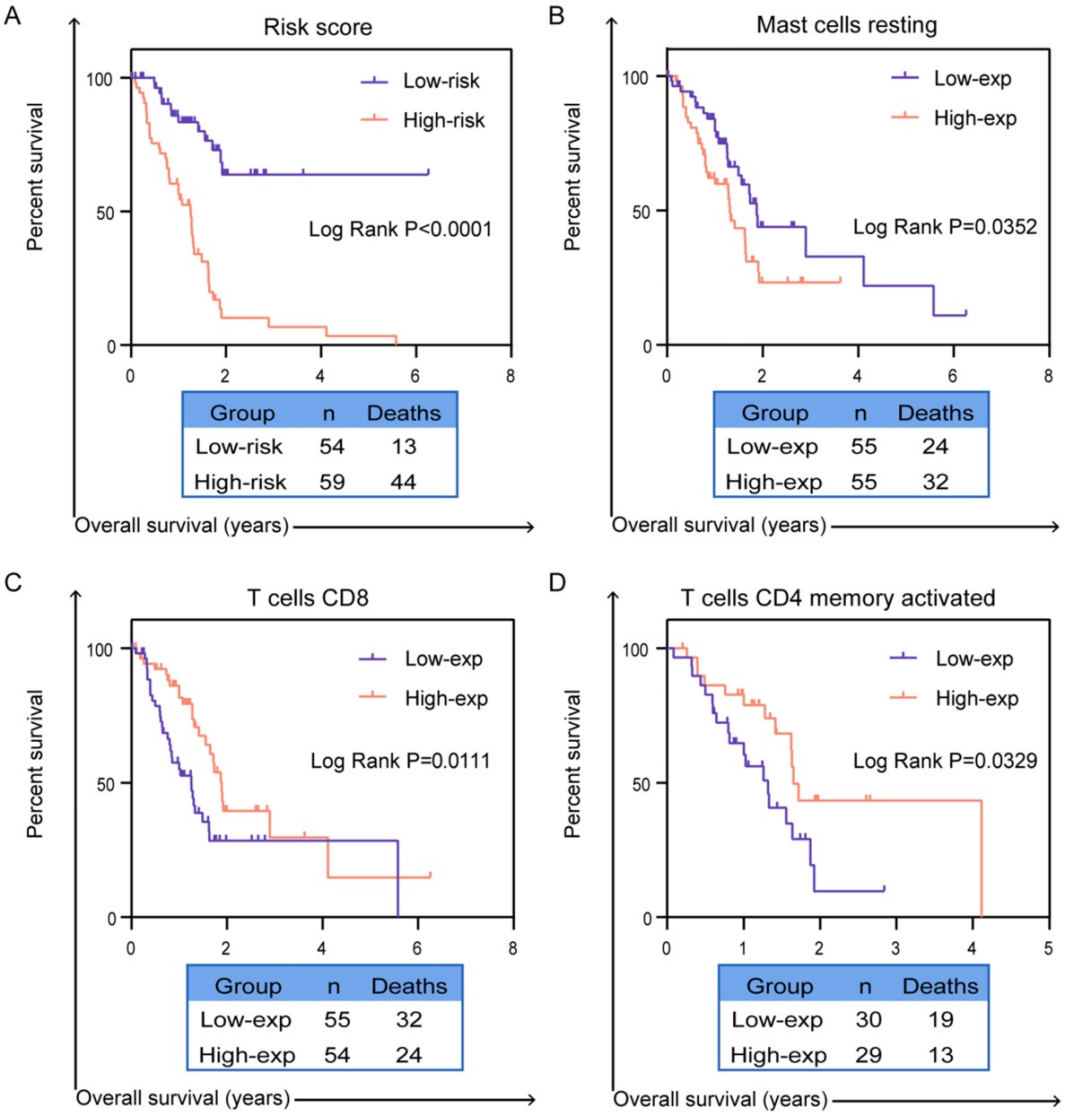
** Validation the prognostic efficiency of infiltrating immune cells in patients.** The Kaplan-Meier survival curves of OS of (**A**) risk score, (**B**) resting mast cell, (**C**) CD8 T cells and (**D**) activated memory CD4 T cells. A two‐sided Log‐Rank *P*<0.05 were considered significant.

More studies has been reported, infiltration of immune cells in tumor microenvironment was accompanied by cancer initiation and progression. Further investigation indicated that the presence of infiltrating immune cells can be used as biomarker for immunotherapy response 19. Therefore, in order to valid whether our prognostic signature in PC patients highlights a correlation between tumor invasion and immune contexture, we applied CIBERSORT algorithm to discuss the proportions of distinct immune cell types with gene expression profiles from TCGA-PC. CIBERSORT algorithm can assess the infiltration of different immune cells by assigning different *P*-value to each sample 20. In final, we got a data cohort with 113 PC patients with CIBERSORT* P* ≥ 0.05. As shown in Figure 7A, we firstly tested the percentage of 22 immune cells in each patient with PC. Then, these patients were classified into two (low/high) risk groups based on prognostic signature. Distribution of immune cell-type fractions in low-risk groups compared with high-risk were shown in Bar charts (Figure 7B). In addition, we investigated the correlation between each cell type which showed that naive CD4 T cells were highly correlated with B cells memory in PC patients (Figure 7C). Furthermore, as shown in the results, the fractions of macrophages M1 (*P*=0.0137, Figure 7D) and resting mast cells (*P*=0.0433, Figure 7E) were significantly higher in higher risk group than that one, whereas the fraction of CD8 T cells (*P*=0.0071, Figure 7F), T cells regulatory (*P*=0.0392, Figure 7G), naive B cells (*P*=0.0127, Figure 7H) and memory B cells (*P*=0.0319, Figure 7I) were indeed lower in high-risk group. There indeed have huge differences in immunological filtration composition, which are probably critical factors in clinical immune-targeted therapy and patients' prognosis in PC. Furthermore, univariate and multivariate survival analysis were applied based on the study of the relationship between the proportion of different immune cells in PC patients and their survival (Supplementary Table S2). The results of univariate survival analysis indicated that signature representing risk score, CD8 T cells, activated memory CD4 T cells and resting mast cells which were associated with OS could be prognostic indicators in PC patients. Importantly, risk score and mast cells are more consistently predicted OS than did other signatures in multivariate survival analysis. K-M curves also confirmed that risk score (*P*<0.0001, Figure 8A), CD8 T cells (*P*=0.0111, Figure 8B), activated memory CD4 T cells (*P*=0.0329, Figure 8C) and resting mast cells (*P*=0.0352, Figure 8D) have prognostic benefit for patients with PC. All above results show that the infiltrating immune cells in tumor microenvironment were associated with prognostic signature. What we identified in the study could provide prognostic biomarker for PC patients and eventually for personalized immune-targeted therapy.

## Discussion

PC is one of the critical causes of cancer-related deaths worldwide. Its incidence rate and mortality rate increase year by year, which are obliged to attract the attention of scientists 21. Increasing evidence demonstrated that isomers, formed after AS of genes, can clearly contribute to tumor progression. These genes were proved to be the determinant of scientists' research 22. Splicing events of cancer-related genes have the potential to gain oncogenic activity may potentially be used as candidate genes for prognostic biomarkers in various cancers 23. For example, Kahles et al. systematic analyzed splicing landscape across 8,705 cancer patients 24. Yu and his colleagues identified of differential AS genes by expression profiling in gliomas 25. Novel signature (seven genes) indicated clinical reaction and platinum sensitivity of serous ovarian carcinoma 26. Xie et al. studied prognostic splicing signatures and revealed regulatory network in esophageal carcinoma 27. Similarly in PC, lots of studies revealed the role of statistic aberrant splicing events. For instance, Choi et al. revealed the clinical application of RHAMM isoforms in the process of pancreatic tumor progression 28. In another study, Yang et al. identified of potential AS prognostic indicators in Pancreatic Ductal Adenocarcinoma 29. Moreover, gene of MALT1 with splicing events can activate CD4^+^ T cells 30. In addition, the pattern of hMENA isoforms is regulated by TGF-beta1 in PC and may predict patient outcome 31. Thus, the potential significance of AS in clinical application and tumor biology has been revealed with each passing day. Therefore, it is urgent to identify AS related biomarkers to predict prognosis in PC.

Integrated prognostic splicing biomarker identified in the present study is including UBA1, S100A13, SH3KBP1, COPSTA, GSE1 and NISCH. The signature model has robust effect to predict prognosis in PC patients. According to NCBI, Ensemble and SMART database, UBA1 has been understood as the ubiquitin-activating enzyme E1 which involved in protein homeostasis. Inhibitors of UBA1 represent an effective target spot for cancer therapy 32. Demonstrated in mouse models also showed the E1 inhibitor can regulate the nuclear factor-κB (NF-κB) and tumor suppressor p53, that strongly represented the value of E1 inhibitors as tumor therapeutic drugs 33. S100A13 can act on the regulation of FGF-1 and participate in the regulation of VEGF-A, which is related to tumor grading and promotes tumor to be more invasive and aggressive. S100A13 has been investigated as a new angiogenesis biomarker in human melanoma 34 and astrocytic gliomas 35. SH3KBP1 can encode an adapter protein and its splicing can make multiple transcript variants with distinct function. Study demonstrated that SEPT9 competes with the ubiquitin ligase Cb1 for binding to SH3KBP1 and inhibits ubiquitylation of epidermal growth factor receptors in cells which can induce or support the development of cancers 36. COPSTA has not been identified clearly yet and it should go further study. GSE1 encodes a proline-rich protein with coiled coil domains, can predict poor survival outcome in gastric cancer 37. Previously, it also reported that GSE1 can make breast cancer deteriorate rapidly under the action of upstream mir-489-5p 38. NISCH encodes a non-adrenergic imidazoline-1 receptor protein, which is mainly located in the inner layer of plasma membrane. This protein has different functions in different cancers. The increased expression in human breast cancer can promote tumor metastasis and invasion. The mechanism may be achieved by controlling the expression of α-5 integrin. However, in ovarian cancer, the decreased expression of NISCH can make cancer cells deteriorate rapidly including proliferation and metastasis. 39. Therefore, it is possible to formulate the hypothesis that changes in these genes could be involved with cancer and the role of their splicing events should be investigated in the future.

Studies have been reported that AS events have been involved in a variety of tumor processes, including angiogenesis, invasion and immune destruction 40. Genes-derived splicing events can produce neoepitopes for immunotherapy to improve patients' survival 41. But there is a lack of bioinformatics analysis of alternative splicing and immunity in pancreatic cancer. We first discovered the correlation between alternative splicing and immunity, and revealed differential distribution of immune cells in different groups of patients separated by identified splicing signature. In our study, we found the differential distribution of more immune cells including macrophage M1, resting mast cells, CD8 T cells, regulation of T cells (Tregs), naive B cells and memory B cells in high- and low-risk groups of patients divided by splicing signature. We also found that CD8 T cells, activated memory CD4^+^ T cells and mast cells have prognostic effect in PC. Among them, high densities of mast cells are clearly related to poor prognosis. This view has indeed been reported in the investigation of skin cancers 42 and malignant melanoma 43. In general, our study suggested that there are great correlation between splicing signature and immune cells in PC.

Immunotherapy based on immune cells can provide effective treatment for some previously untreatable cancers including PC. However, one of the difficulties and urgency in the immunotherapy is to search appropriate target antigens 44. Notably, in the process of searching for new antigens of tumor mutations, neoantigen epitopes produced by mRNA splicing events has been paid enough attention. Recently, study has shown that peptides produced by mRNA splicing events have the potential to bind to MHC class I molecules where they serve as neoepitopes 45. So hypothetically the splicing events identified in our study can generate neoepitopes for CD8^+^ or CD4^+^ T cells, cancer immunotherapy targets will be largely expand. In terms of clinical sense, using the neoantigen peptide to prepare personalized vaccine and inject it into patients to produce T cells responses. This will relieve or eliminate cancer cells and improve patients' prognosis.

## Conclusions

In conclusion, we demonstrated the robust prognostic value of AS events in PC, which can provide basis for clinical application. Furthermore, we indeed provide novel insights into the correlation between AS and immune cells. This result has far-reaching significance for immunotherapy in the future. Moving forward, the profound study of splicing events and immune cells can indeed represent novel tumor drivers and provide potential targets for personalized therapeutic intervention.

## Supplementary Material

Supplementary figures and tables.

## Figures and Tables

**Table 1:** Summary of clinical characteristics of PC patients in entire TCGA set (n=175), TCGA validation set 1 (n=88) and TCGA validation set 2 (n=87)

Characteristic	Patients in entire TCGA set (n=175), n (%)	Patients in validation set 1 (n=88), n (%)	Patients in validation set 2 (n=87), n (%)
**Age (years)**			
<65	87 (49.71)	47 (53.41)	35 (40.23)
≥65	88 (50.29)	41 (46.59)	52 (59.77)
**Gender**			
Female	80 (45.71)	42 (47.73)	38 (43.68)
Male	95 (54.29)	46 (52.27)	49 (56.32)
**History type**			
PAAD	145 (83.43)	71 (80.68)	74 (85.06)
Other subtype	29 (16.00)	17 (19.32)	12 (13.79)
NA	1 (0.57)	0 (0.00)	1 (1.15)
**Vital status**			
Alive	87 (49.71)	39 (44.32)	44 (50.57)
Dead	88 (50.29)	49 (55.68)	43 (49.43)
**Cancer stage**			
Stage I	21 (12.00)	14 (15.91)	7 (8.05)
Stage II	144 (82.29)	71 (80.68)	73 (83.91)
Stage III-IV	7 (4.00)	2 (2.27)	5 (5.75)
NA	3 (1.71)	1 (1.14)	2 (2.30)
**Race**			
Asian	10 (5.71)	3 (3.41)	7 (8.05)
Black or African American	6 (3.43)	0 (0.00)	6 (6.90)
White	155 (88.57)	82 (93.18)	73 (83.91)
NA	4 (2.29)	3 (3.41)	1 (1.15)
**Pathological stage N**			
N0	49 (28.00)	20 (22.73)	29 (33.33)
N1	121 (69.14)	64 (72.73)	57 (65.52)
NA	5 (2.86)	4 (4.55)	1 (1.15)
**Pathological stage T**			
T1-T2	31 (17.71)	21 (23.86)	10 (11.49)
T3-T4	142 (81.14)	66 (75.00)	76 (87.36)
NA	2 (1.14)	1 (1.14)	1 (1.15)
**Pathological stage M**			
M0	78 (44.57)	41 (46.59)	37 (42.53)
M1	4 (2.29)	1 (1.14)	3 (3.45)
NA	93 (53.14)	46 (52.27)	47 (54.02)
**Cancer Status**			
Tumor Free	54 (30.86)	26 (29.55)	28 (32.18)
With Tumor	71 (40.57)	40 (45.45)	31 (35.63)
NA	50 (28.57)	22 (25.00)	28 (32.18)
**Grade**			
G1	30 (17.14)	19 (21.59)	11 (12.64)
G2	95 (54.29)	42 (47.73)	53 (60.92)
G3-G4	49 (28.00)	26 (29.55)	23 (26.44)
NA	1 (0.57)	1 (1.14)	0 (0.00)
**New event**			
No	70 (40.00)	32 (36.36)	38 (43.68)
Yes	105 (60.00)	56 (63.64)	49 (56.32)
**Radiation therapy**			
No	101 (57.71)	51 (57.95)	50 (57.47)
Yes	31 (17.71)	16 (18.18)	15 (17.24)
NA	43 (24.57)	21 (23.86)	22 (25.29)
**History of alcohol**			
No	63 (36.00)	30 (34.09)	33 (37.93)
Yes	100 (57.14)	54 (61.36)	46 (52.87)
NA	12 (6.86)	4 (4.55)	8 (9.20)
**Anatomic neoplasm subdivision**			
Head of pancreas	136 (77.71)	70 (79.55)	66 (75.86)
Body of pancreas	14 (8.00)	6 (6.82)	8 (9.20)
Tail of pancreas	14 (8.00)	8 (9.09)	6 (6.90)
NA	11 (6.29)	4 (4.55)	7 (8.05)
**History of diabetes**			
No	106 (60.57)	61 (69.32)	45 (51.72)
Yes	38 (21.71)	16 (18.18)	22 (25.29)
NA	31 (17.71)	11 (12.50)	20 (22.99)

**Abbreviations:** PC: pancreatic cancer; TCGA: The Cancer Genome Atlas; PAAD: pancreatic adenocarcinoma; NA: not available.

**Table 2:** Univariable and multivariable Cox regression analyses for risk score and different clinical pathological parameters in PC patients

Clinical feature	Number (n)	Univariable analysis			Multivariable analysis		
		HR	95% CI	*P*-value	HR	95% CI	*P*-value
Risk score (Low risk/High risk)	88/87	4.917	2.949-8.199	**<0.0001**	4.746	2.135-10.551	**<0.0001**
Cancer status (Tumor free/with tumor)	54/71	3.405	1.882-6.162	**<0.0001**	1.804	0.724-4.496	0.206
History type (PAAD/other subtype)	146/28	3.095	1.488-6.439	**0.003**	1.404	0.462-4.270	0.550
Pathological stage-T (T1+T2/T3+T4)	31/142	2.272	1.169-4.416	**0.016**	1.224	0.434-3.454	0.703
Pathological stage-N (N0 / N1)	49/121	2.217	1.301-3.780	**0.003**	1.262	0.593-2.685	0.546
Cancer stage (Stage I/II/III-IV)	21/144/7	1.689	1.004-2.840	**0.048**	1.077	0.350-3.308	0.897
Grade (G1/G2/G3-G4)	30/95/97	1.505	1.095-2.069	**0.012**	1.620	1.052-2.496	**0.028**
New event (No/Yes)	70/105	2.257	1.377-3.699	**0.001**	1.638	0.631-4.256	0.311
Radiation therapy (No/Yes)	101/31	2.708	1.339-5.476	**0.006**	1.799	0.754-4.294	0.186

**Abbreviations:** PC: pancreatic cancer; HR: hazard ratio; 95%CI: 95% confidence interval.

## Abbreviations

PC: pancreatic cancer
AS: alternative splicing
TCGA: The Cancer Genome Atlas
PSI: percent spliced index
OS: overall survival
ES: exon skip
AA: alternate acceptor site
AP: alternate promoter
AD: alternate donor site
AT: alternate terminator
ME: mutually exclusive exons
RI: retained intron
GO: Gene Ontology
KEGG: Kyoto Encyclopedia of Genes and Genomes
K-M: Kaplan-Meier
ROC: receiver operating characteristic curve

## Biographies

**Minjie Wei**, PhD, the dean of the school of pharmacy, China Medical University and the director of the pharmacology teaching and research office. She is the leader of pharmacology, a hundred talents project in Liaoning Province, and an honorary professor of Queen's University. The leading talents of the "ten thousand talents plan" of the Organization Department of the CPC Central Committee, specially appointed professors, outstanding experts and famous teachers of Liaoning Province. She is mainly engaged in the research of molecular tumor pharmacology, neuroimmunological pharmacology and pharmacogenomics. She has successively presided over more than 30 national, ministerial, provincial and other scientific research projects, and published more than 200 papers in international and domestic academic journals.

**Lin Zhao**, PhD, Department of Pharmacology, China Medical University, member of Tumor Pharmacology, Chinese Pharmacology Society. She was selected into the "one million talents project" of Liaoning Province. She is mainly engaged in the research of tumor pathogenesis and drug resistance mechanism, and develops innovative drugs for tumor specific targets. She has published more than 40 research papers, 20 of which are included in SCI. She undertook many national and provincial projects and the main finisher of scientific and technological achievements of the ministry of education and scientific and technological awards of Liaoning province. She has applied for 3 patents for research achievements.

**Miao He**, PhD, Department of Pharmacology, China Medical University, In 2003, She worked in China Medical University, and from 2008 to 2010, she conducted postdoctoral research in the cancer research section of the medical center of the University of Chicago. At present, she is the deputy director of pharmacology teaching and research office. She is mainly engaged in the research on the pathogenesis and drug resistance mechanism of breast cancer, ovarian cancer and other tumors, especially the research on drug resistance mechanism mediated by tumor stem cells. She has undertaken 2 National Natural Science Fund projects, 1 Liaoning Natural Science Fund funded project, 1 Liaoning University outstanding young scholars growth plan project, and 5 Liaoning University students innovation and entrepreneurship projects. She has published more than 60 research papers, including more than 30 papers included in SCI.
