# Supplementary material for: Prognostic alternative splicing signature reveals the landscape of immune infiltration in Pancreatic Cancer
Source: J Cancer. 2020 Sep 21;11(22):6530–44. doi: 10.7150/jca.47877 (PMC7545682; doi:10.7150/jca.47877)
Supplement: Supplementary file 1 — Supplementary figures and tables. [file jcav11p6530s1.pdf]

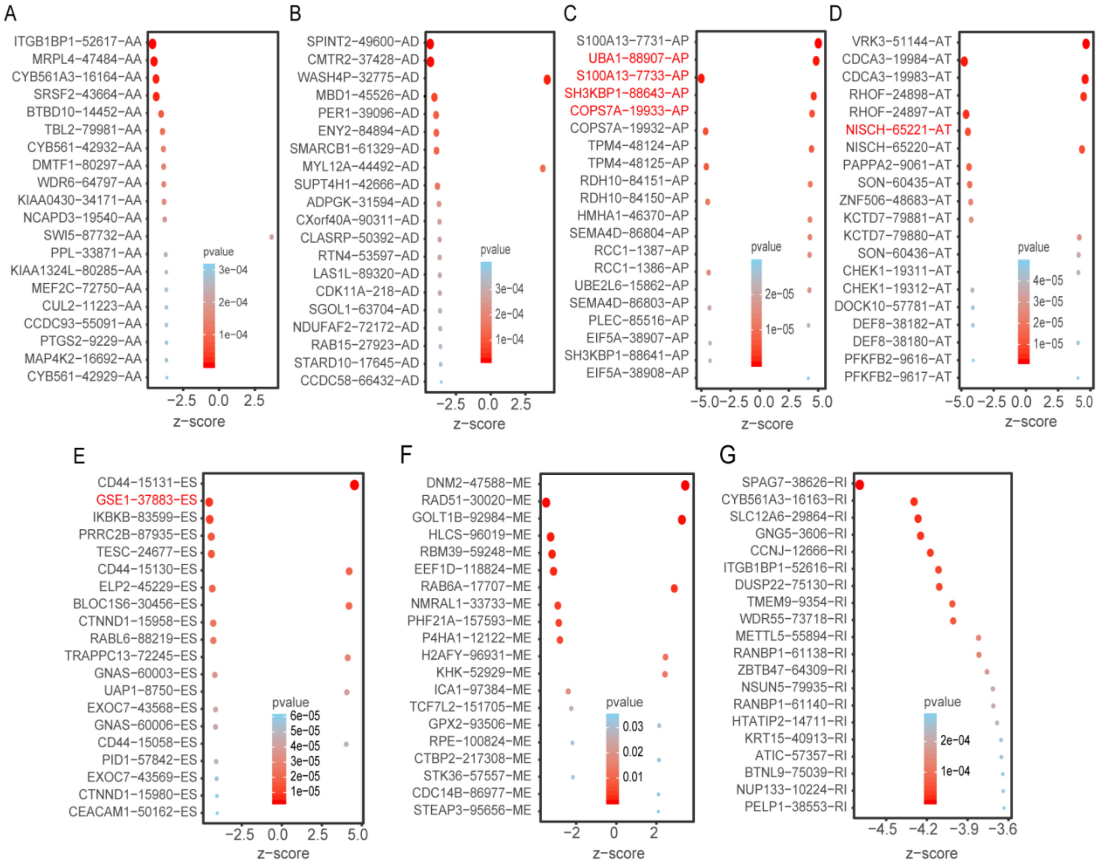

Supplementary Figure S1 The differentially survival-associated AS events between patients with PC and normal group. The top 20 significantly survival-related AS genes and events in seven AS types including (A) AA (B) AD (C) AP (D) AT (E) ES (F) ME (G) RI in PC patients. The highlight red genes were those which included in identified prognostic signature. **Abbreviation:** AA: alternate acceptor site; AD: alternate donor site; AP: alternate promoter; AT: alternate terminator; ES: exon skip; ME: mutually exclusive exons; RI: retained intron.

A

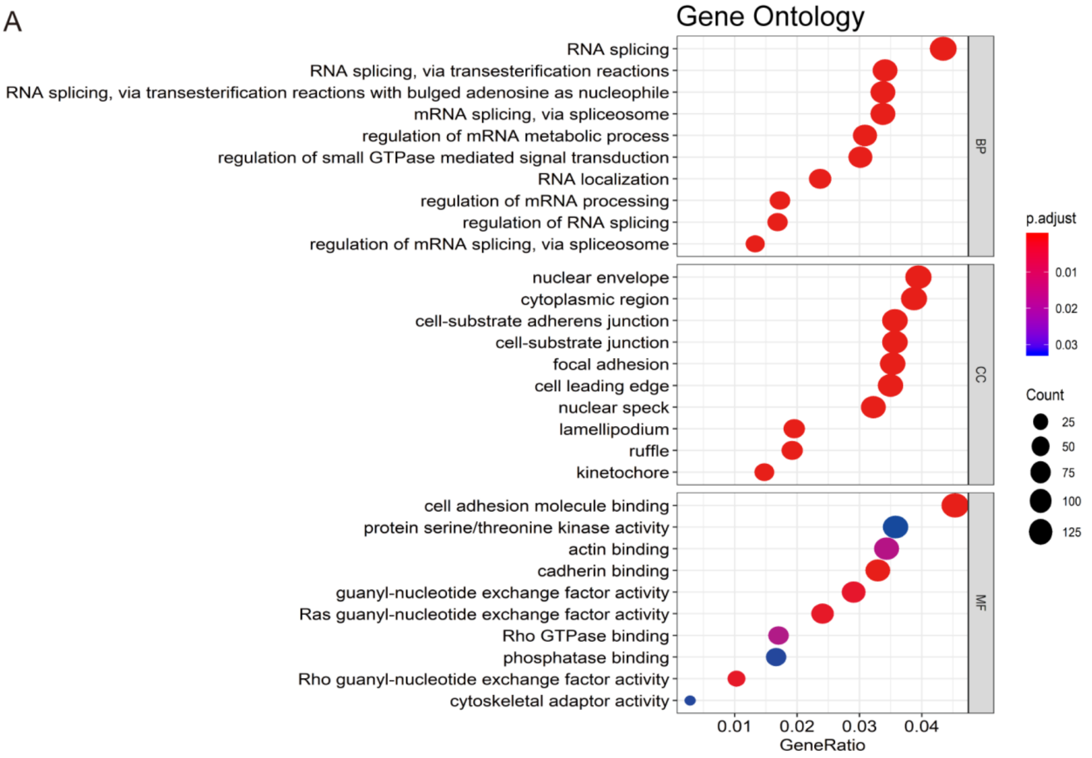

B

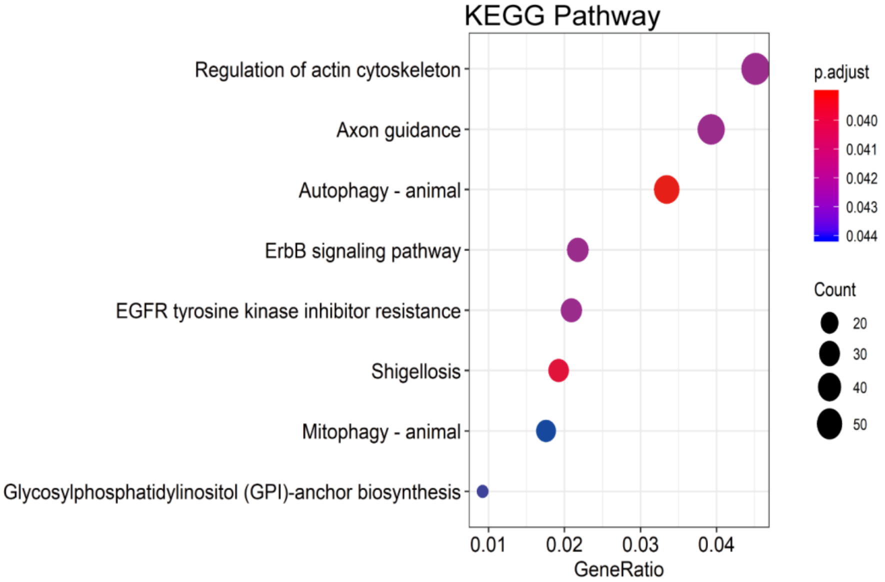

Supplementary Figure S2 Functional enrichment analysis of differentially AS genes in PC. (A) Significantly enriched Gene Ontology terms of differentially AS genes in PC. BP, biological process; CC, cellular component; MF, molecular function. (B) Kyoto Encyclopedia of Genes and Genomes enriched pathways of differentially splicing genes in PC.

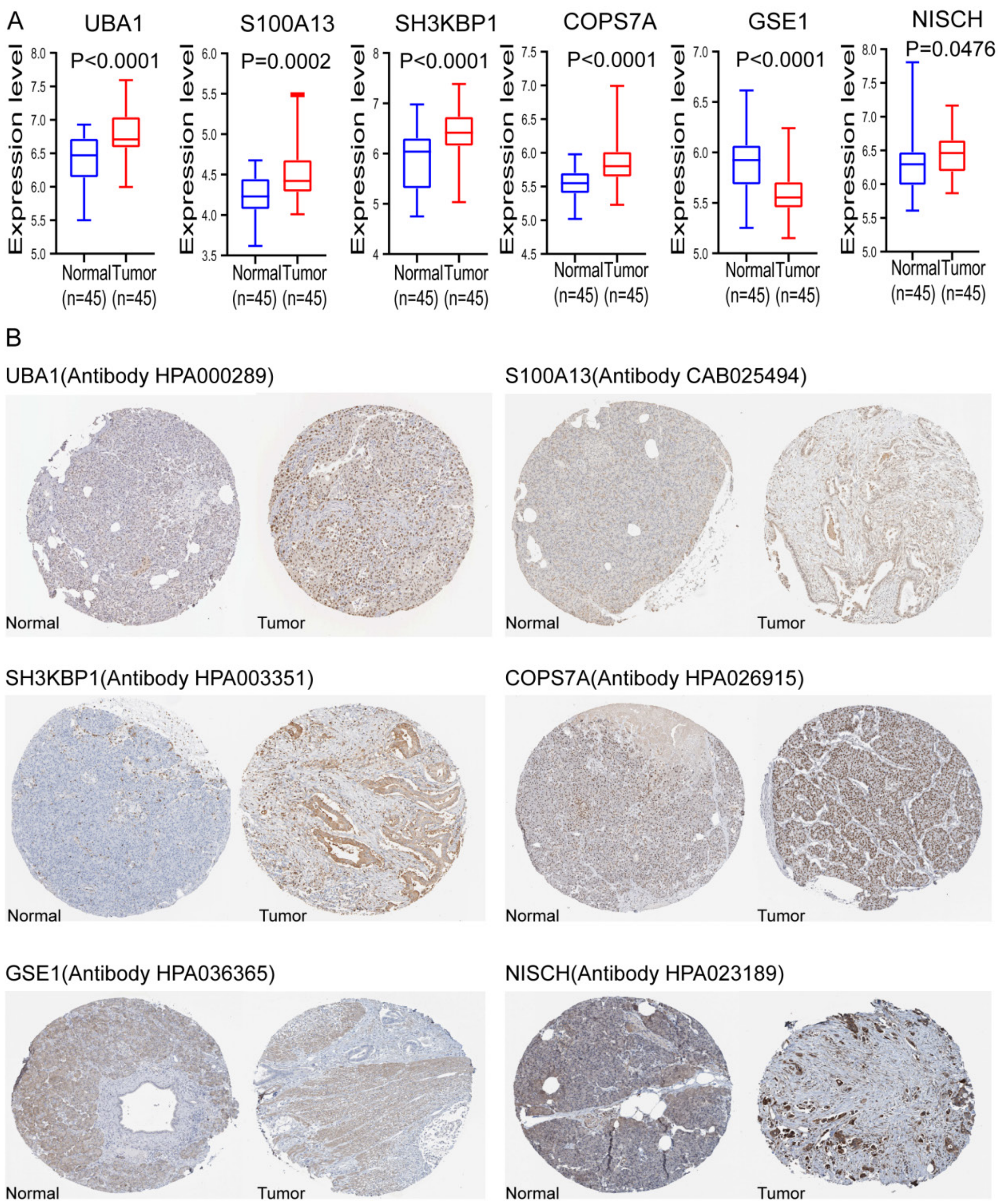

Supplementary Figure S3 Differential expressed validation of six genes of splicing signature in different database. (A) Differential expressed analysis of six genes between normal and tumor in GSE28735. (B) Validation of the differential expression of six proteins between normal and PC tissues in HPA database. UBA1 (antibody HPA000289) presented high expression in tumor tissue (staining intensity: strong; quantity: >75%) and low expression in normal tissue (staining intensity: weak; quantity: 75%-25%). S100A13 (antibody CAB025494) presented medium expression in tumor tissue (staining intensity: moderate; quantity: >75%) and low expression in normal tissue (staining intensity: weak; quantity: <25%). SH3KBP1 (antibody HPA003351) presented medium expression in tumor tissue (staining intensity: moderate; quantity: >75%) and negative in normal tissue (staining intensity: negative). COPS7A (antibody HPA026915) presented high expression level both in tumor and normal tissue (staining intensity: strong; quantity: >75%). GSE1 (antibody HPA036365) was negative in tumor tissue (staining intensity: negative) and low expression in

normal tissue (staining intensity: weak; quantity: 75%-25%). NISCH (antibody HPA023189) presented high expression in tumor tissue (staining intensity: strong; quantity: >75%) and medium expression in normal tissue (staining intensity: moderate; quantity: <25%). **Abbreviations:** PC: pancreatic cancer; HPA: Human Protein Atlas.

Supplementary Table S1 The detailed information of six alternative splicing events significantly related to overall survival in PC patients (n=175).

ID	EXP(βi)	HR	95%CI	*P*-value
UBA1|AP	5.5797	264.9938	1.8155-38678.5840	0.0282
S100A13|AP	-4.1249	0.0162	0.0004-0.6565	0.0291
SH3KBP1|AP	1.2863	3.6193	1.1351-11.5397	0.0297
COPS7A|AP	2.8034	16.5012	2.4428-111.4641	0.0040
GSE1|ES	-1.5478	0.2127	0.0537-0.8423	0.0275
NISCH|AT	-3.5896	0.0276	0.0011-0.7228	0.0312

Abbreviations: PC: pancreatic cancer; AP: alternate promoter; ES: exon skip; AT: alternate terminator; HR: hazard ratio; 95%CI: 95% confidence interval.

Supplementary Table S2 Univariable and multivariable Cox regression analyses of infiltrated immune cells to identify prognostic factors in PC patients.

Variables	Univariate analysis			Multivariate analysis		
	HR	95%CI	*P*-value	HR	95%CI	*P*-value
Risk score	5.000	2.664-9.387	**<0.0001**	4.572	1.905-10.972	**0.001**
T cells CD8	0.506	0.296-0.864	**0.013**	0.494	0.222-1.098	0.083
T cells CD4 memory activated	0.461	0.222-0.956	**0.037**	0.463	0.212-1.010	0.053
Mast cells resting	1.783	1.033-3.078	**0.038**	2.230	1.013-4.908	**0.046**
T cells follicular helper	1.889	0.822-4.341	0.134	-	-	-
Macrophages M0	1.499	0.872-2.575	0.143	-	-	-
Macrophages M1	1.505	0.871-2.602	0.143	-	-	-
B cells memory	0.494	0.191-1.280	0.146	-	-	-
T cells regulatory (Tregs)	0.672	0.383-1.180	0.167	-	-	-
Monocytes	0.756	0.388-1.472	0.411	-	-	-
Dendritic cells activated	1.201	0.546-2.641	0.648	-	-	-
NK cells activated	0.925	0.520-1.644	0.790	-	-	-
T cells CD4 memory resting	1.075	0.626-1.845	0.794	-	-	-
Macrophages M2	1.054	0.626-1.776	0.842	-	-	-
Neutrophils	0.942	0.475-1.868	0.864	-	-	-
B cells naive	0.949	0.519-1.738	0.866	-	-	-
Plasma cells	0.969	0.527-1.781	0.919	-	-	-

Abbreviations: PC: pancreatic cancer; HR: hazard ratio; 95%CI: 95% confidence interval.

NOTE: “-” represents that corresponding factors not included in the multivariable analysis.
